# Is IL-1β Further Evidence for the Role of *Propionibacterium acnes* in Degenerative Disc Disease? Lessons From the Study of the Inflammatory Skin Condition Acne Vulgaris

**DOI:** 10.3389/fcimb.2018.00272

**Published:** 2018-08-14

**Authors:** Ondrej Slaby, Andrew McDowell, Holger Brüggemann, Assaf Raz, Sibel Demir-Deviren, Tony Freemont, Peter Lambert, Manu N. Capoor

**Affiliations:** ^1^Central European Institute of Technology, Masaryk University, Brno, Czechia; ^2^Northern Ireland Centre for Stratified Medicine, School of Biomedical Sciences, Ulster University, Londonderry, United Kingdom; ^3^Department of Biomedicine, Aarhus University, Aarhus, Denmark; ^4^Laboratory of Bacterial Pathogenesis and Immunology, Rockefeller University, New York, NY, United States; ^5^Spine Center, UCSF Medical Center, San Francisco, CA, United States; ^6^Division of Cell Matrix Biology and Regenerative Medicine, Faculty of Biology Medicine and Health, The University of Manchester, Manchester, United Kingdom; ^7^School of Life and Health Sciences, Aston University, Birmingham, United Kingdom

**Keywords:** *Propionibacterium acnes*, interleukin-1 beta, nerve growth factor (NGF), degenerative disc disease, acne vulgaris

## Abstract

The pathogenesis of degenerative disc disease is a complex and multifactorial process in which genetics, mechanical trauma, altered loading and nutrition present significant etiological factors. Infection of the intervertebral disc with the anaerobic bacterium *Propionibacterium acnes* is now also emerging as a potentially new etiological factor. This human commensal bacterium is well known for its long association with the inflammatory skin condition acne vulgaris. A key component of inflammatory responses to *P. acnes* in acne appears to be interleukin (IL)-1β. Similarly, in degenerative disc disease (DDD) there is compelling evidence for the fundamental roles of IL-1β in its pathology. We therefore propose that *P. acnes* involvement in DDD is biologically very plausible, and that IL-1β is the key inflammatory mechanism driving the host response to *P. acnes* infection. Since there is a solid theoretical basis for this phenomenon, we further propose that the relationship between *P. acnes* infection and DDD is causal.

## The clinical problem and epidemiological evidence of *Propionibacterium acnes* in degenerative disc disease

Chronic low back pain (CLBP) is the leading cause of disability/worker's compensation claims, second leading cause for patient-doctor visits, the third most common cause of surgical procedures, and the fifth-ranking cause of admission to hospital (Hart et al., 1995). Although many conditions lead to CLBP, degenerative disc disease (DDD) is among the most common diagnoses. Because correlations among clinical symptoms, radiological signs of disc degeneration, and treatment outcomes are not satisfactory, the selection of treatment options is difficult and the options employed often yield less than satisfactory results. Clearly, a more detailed understanding of the causes and development of DDD is needed so patients can be stratified for tailored or personalized treatments where possible.

The identification of *Propionibacterium acnes* biofilms in intervertebral disc tissue obtained from patients who have undergone microdiscectomy, combined with previous studies demonstrating the success of antibiotic treatment for patients with CLBP (Albert et al., 2013), has led to the suggestion that *P. acnes* infection of the intervertebral disc may be another important causative factor for the development of DDD. The mechanisms by which *P. acnes* infection may lead to DDD have, however, yet to be elucidated. Here, we propose a model of *P. acnes* pathogenicity in DDD in which patients may benefit from biomarker-directed anti-inflammatory interventions, such as already available biologic drugs or small molecule inhibitors that could be re-purposed for a more precision-based approach to treatment, in combination with antibiotic or other antimicrobial treatments.

**Figure 1:**
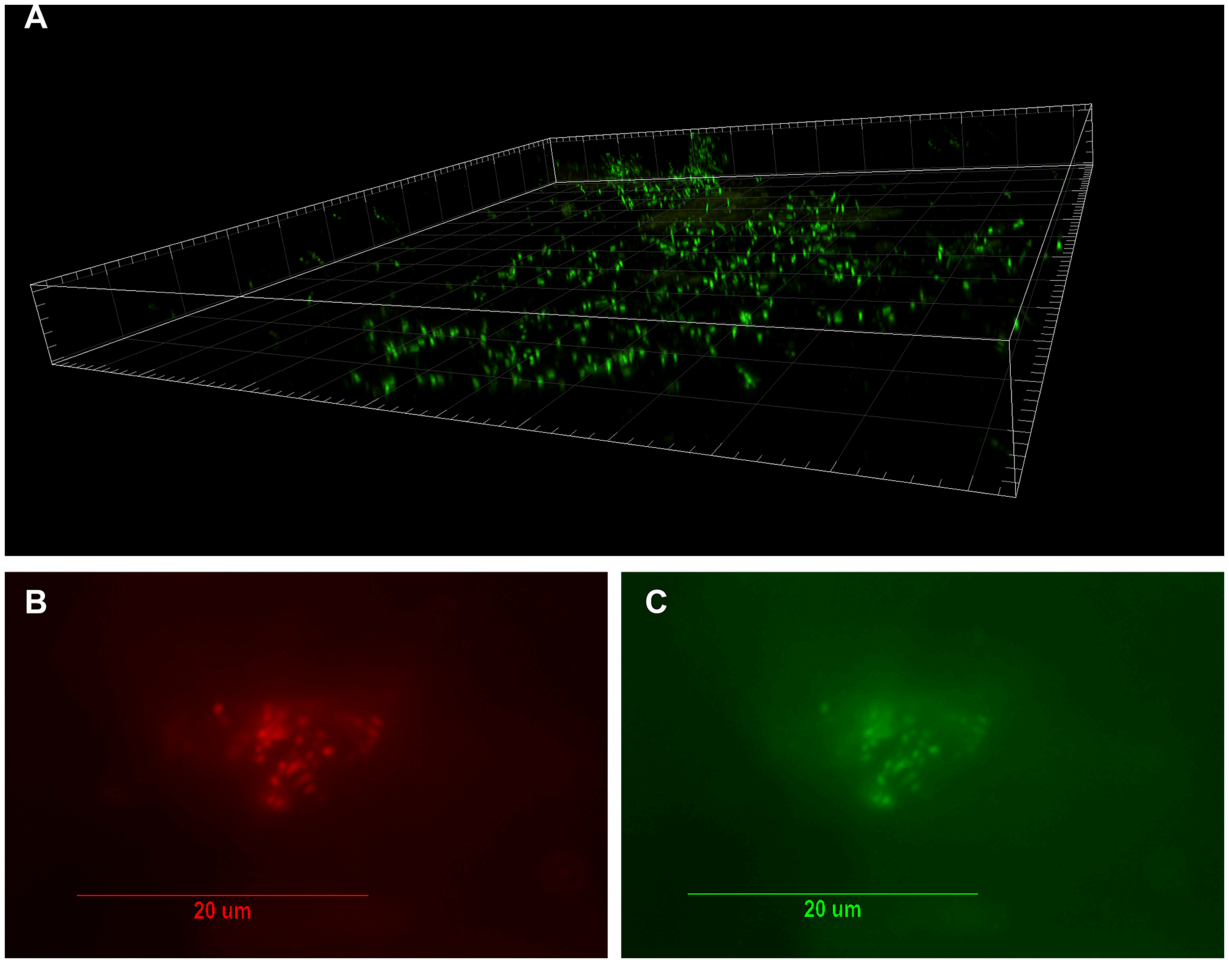
Visualization of bacterial biofilm in the disc tissue by confocal laser scanning microscopy (CSLM) and confirmation of *P. acnes* by fluorescence *in-situ* hybridization (FISH) (reprinted from Capoor et al., 2017). **(A)** Three dimensional reconstructed CSLM image of biofilm bacteria stained with a DNA stain (SYTO9, green) in a disc tissue sample. **(B,C)** The presence of *P. acnes* biofilms in this sample verified using FISH. Epifluorescence micrographs of a biofilm cluster showing red fluorescence from the CY5-labeled EUB338 general eubacterial probe **(B)** and green fluorescence from the CY3-labled *P. acnes*-specific probe **(C)** Co-localization of the red and green fluorescence indicates that all of the bacteria in this biofilm were *P. acnes*.

**Figure 2:**
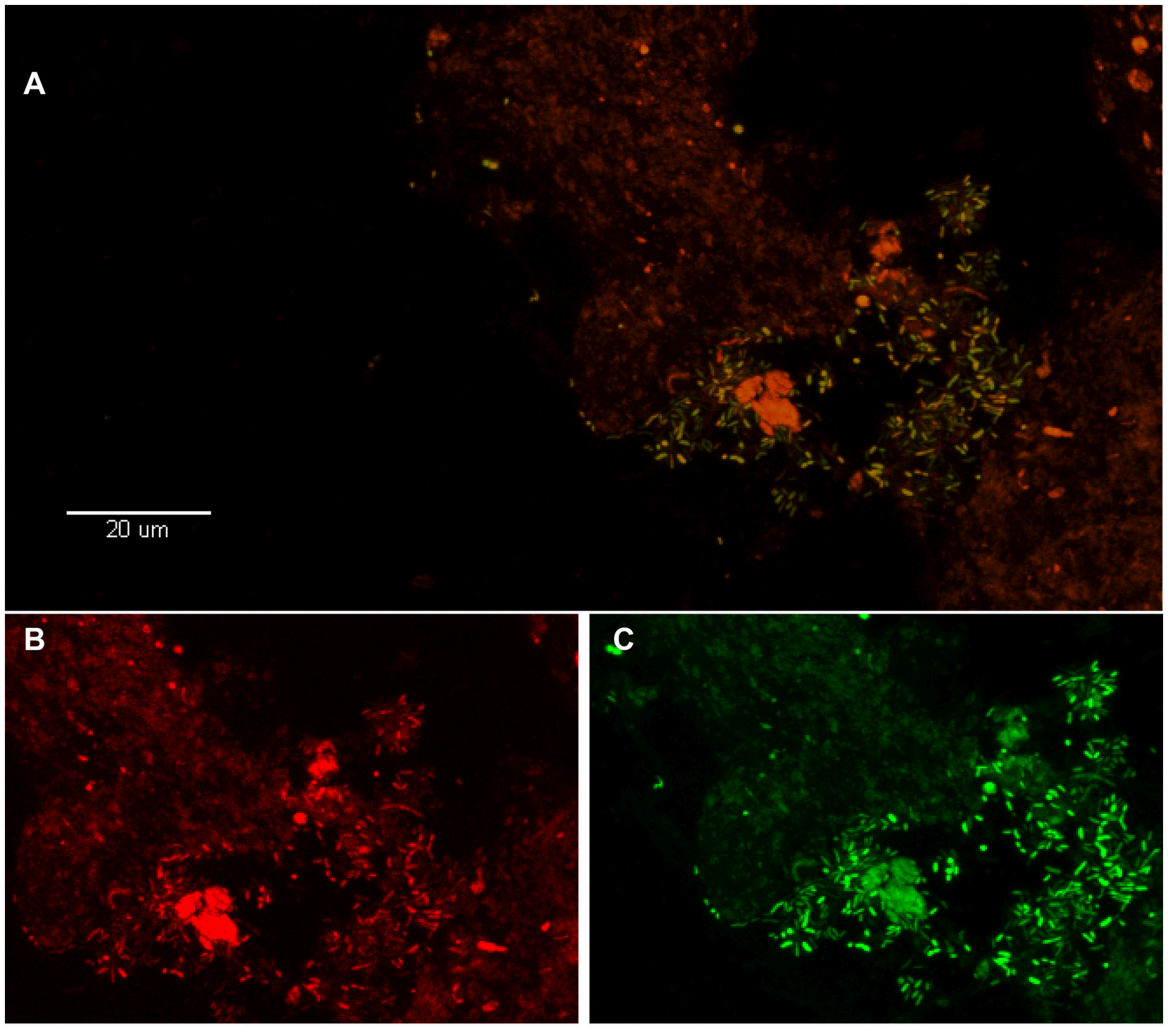
Visualization of *P. acnes* biofilm in the disc tissue by use of FISH (reprinted from Capoor et al., 2017). **(A)** This color-combined image shows the “pocket” of green fluorescent *P. acnes* cells (biofilm) near the center right of the image. The presence of *P. acnes* biofilms in this sample was verified using FISH. **(B,C)** Red fluorescence is the general eubacterial probe **(B)** and green is the *P. acnes* probe **(C)**. The **(B,C)** image is a zoom of **(A)** showing fluorescence from the red and green channels separately. Almost all of the cells in **(A)** are emitting both red and green fluorescence, indicating that they are *P. acnes*.

The seminal, potentially paradigm-shifting study that first provided evidence to link *P. acnes* infection of the degenerated disc to CLBP was published by Stirling et al. (2001). Since then, the role of this bacterium in CLBP has received considerable attention, as well as controversy, due to the possibility of novel treatment options for this debilitating condition (Birkenmaier, 2013). Subsequent independent research into this exciting area has been collectively examined in two independent meta-analyses published in 2015. These studies reported that the pooled prevalence of bacterial infection was 34% (Urquhart et al., 2015) or 36.2% (Ganko et al., 2015), and confirmed *P. acnes* as the major bacterial species present in retrieved disc samples. Very recently, *P. acnes* was further identified as the only significant bacterial organism present in degenerated disc tissue obtained from 32% of 368 patients undergoing microdiscectomy (Capoor et al., 2017). Importantly, this study also provided the first microscopic evidence of *P. acnes* biofilm within degenerated disc tissue as shown in Figures 1, 2 (Capoor et al., 2017). The presence of biofilm, which is consistent with colonization/ infection rather than perioperative contamination, which is a key concern when dealing with an organism which also forms part of the endogenous microbiota, provided compelling evidence that at least some of the culture-positive degenerated disc tissue had truly become infected with *P. acnes* (Capoor et al., 2017). Both rabbit and rat models have also shown that *P. acnes* infection of disc tissue can induce disc degeneration, inflammatory response in the endplate region and MC1-like changes in the adjacent bone marrow (Dudli et al., 2016; Shan et al., 2017).

## A key role for IL-1β in *P. acnes* driven acne vulgaris

The involvement of *P. acnes* in acne was first suggested as early as 1896 when Unna isolated the bacterium from an acne lesion (Unna, 1895). Even since then, the exact role the organism plays in the initiation and progression of this chronic inflammatory skin condition remains unclear, especially as some inflamed lesions show no evidence of viable bacterial colonization and no correlation exists between bacterial counts and severity of inflammation (Shaheen and Gonzalez, 2011). Acne samples do, however, demonstrate a higher prevalence of follicular *P. acnes* colonization, both in terms of follicles containing *P. acnes* and the greater numbers of bacteria in macrocolonies/biofilms than in control samples (Jahns et al., 2012). The strong inflammatory potential of *P. acnes* is well known, and the bacterium can upregulate a wide range of inflammatory cytokines (e.g., TNFα, IL-6, IL-8, IL-1 family) and activate keratinocytes and sebocytes via CD1, CD14, and Toll-like receptors (TLRs) (Moradi Tuchayi et al., 2015). The bacterium can also induce the expression of IL-17 from peripheral blood mononuclear cells and promote Th17 and Th1 response pathways (Agak et al., 2014); activation of the Th17 axis and Th17 cytokines (IL-17, IL-1β, IL-6, and TGF-β) appears to play a pivotal role in acne (Sardana and Verma, 2017). Furthermore, the organism produces lipases which facilitate breakdown of the sebum (carbon source) into free short chain fatty acids which are pro-inflammatory (Sanford et al., 2016). The critical role of the IL-1 family of cytokines in the pathogenesis of acne vulgaris, especially IL-1β which is abundant in acneic lesions, has now been well described (Kistowska et al., 2014). This strongly pro-inflammatory cytokine has been associated with IL-17 production highlighting the strong link between IL-1β and IL-17 immunity (Lasigliè et al., 2011).

The induction of IL-1β is believed to be a key driver of inflammatory responses in acne and is mediated via inflammasome activation (Kistowska et al., 2014). We can easily envisage, therefore, how *P. acnes* pathogen-associated molecular patterns (PAMPs) will bind to TLR2 on sebocytes (Li et al., 2014) and myeloid cells (Kistowska et al., 2014), activating NF-κB signaling and consequently transcription of the IL-1β gene, thus leading to the production of pro-IL-1β within the pilosebaceous unit. This initial interaction between *P. acnes* and myeloid cells can occur either by migration of the latter into the follicular space or by leakage of *P. acnes* into the peri-follicular space. Myeloid cells and sebocytes constitutively express inflammasome proteins. *P. acnes* PAMPs trigger NLRP3 inflammasome activation, resulting in increased caspase 1 activity, facilitated cleavage of pro-IL-1β, and excessive release of mature IL-1β. Furthermore, IL-1β promotes dermal matrix destruction by inducing proteolytic matrix metalloproteases (MMPs). Progression of the inflammation results in *P. acnes* overgrowth and follicular obstruction, which provokes follicular rupture. *P. acnes* can then leak out of the pilosebaceous unit and get into close contact with the peri-follicular myeloid cells, finally leading to excessive release of IL-1β and neutrophil-rich peri-follicular inflammation.

## A key role for IL-1β in degenerative disc disease

Disc degeneration is also known to be mediated by the abnormal secretion of pro-inflammatory cytokines by the inner nucleus pulposus (NP) and outer annulus fibrosus (AF) cells of the intervertebral disc, as well as by immune cells attracted to the site of disc degeneration. These cytokines, including IL-1α, IL-1β, IL-6, IL-8, IL-17, TNF-α and IFN-γ, serve to promote a range of pathogenic responses by disc cells including matrix breakdown and immune cell recruitment (Risbud and Shapiro, 2014). As with acne vulgaris, IL-1β appears to play a key role in disc degeneration, including matrix destruction, angiogenesis and cellular apoptosis and senescence (Yang et al., 2015). Levels of the cytokine in degenerated discs also appear to correlate with the degree of degeneration (Le Maitre et al., 2005). Also, IL-17 expression is increased in degenerated discs as well as intervertebral disc cells exposed to IL-1β (Le Maitre et al., 2005; Yang et al., 2015). As in acne, we see an infiltration of CD68+ macrophages, neutrophils and T cells (CD4+; CD8+) in herniated discs (Risbud and Shapiro, 2014).

Irrespective of the initiating factor in DDD, it is thought to trigger NF-κB signaling through TLRs, which stimulates the production of pro-IL-1β (Risbud and Shapiro, 2014). Increased levels of NLRP3 and caspase-1 have also been described in degenerated disc tissue, indicating inflammasome activation and facilitated maturation of pro-IL-1β (Chen et al., 2015). Inflammatory actions are further amplified because IL-1β is not only an NF-κB target gene, but also an NF-κB activator, forming a positive feedback loop (Chen et al., 2015). This IL-1β/IL-1 receptor (IL-1R) signaling promotes extracellular matrix degradation through the induction of proteolytic enzymes, including MMPs 1, 2, 3, 9, and 13 and aggrecanases of “a disintegrin and metalloproteinase with thrombospondin motifs' (ADAMTS) families 4/5 (Le Maitre et al., 2007). Specific damage-associated molecular patterns (DAMPs), which include fragmented collagen, aggrecan, or hyaluronic acid, bind to TLRs on NP and AF cells and activate NF-κB, forming another positive feedback loop (Quero et al., 2013). IL-1β also promotes the production of chemotactic CC-chemokines, mainly CCL3 and CCL4, leading to the recruitment and activation of infiltrating immune cells that further amplify the inflammatory cascade (Wang et al., 2013). These pathways form the core of a model in which *P. acnes* infection causes IL-1β release by NP, AF and immune cells, which leads to extracellular matrix degradation within the intervertebral disc (Nazipi et al., 2017).

The ingrowth of nociceptive nerve fibers into the degenerated disc, usually accompanied by the presence of annular fissures, is considered a main source of nociception related to CLBP (Freemont et al., 1997). IL-1β has been shown to induce the expression of neurotrophin-like nerve growth factor (NGF) and brain-derived neurotrophic factor (BDNF) in both disc and immune cells, supporting nerve ingrowth into the degenerated disc (Gruber et al., 2012). IL-1β-stimulated NGF and BDNF production further induces expression of pain-associated cation channels in the dorsal root ganglion (Risbud and Shapiro, 2014), the depolarization of which is likely to promote low back and radicular pain. Finally, NGF has a direct activating or sensitizing effect on nociceptors, and its upregulation in CLBP has been demonstrated (Denk et al., 2017).

## Hypothesis: the central role of IL-1β in DDD is evidence of a *P. acnes*-driven etiology

Based on the (i) compelling epidemiological evidence (Stirling et al., 2001; Urquhart et al., 2015; Capoor et al., 2016, 2017; Coscia et al., 2016), (ii) the uniqueness of *P. acnes* in the degenerated disc tissue (Capoor et al., 2017), (iii) direct microscopic evidence of *P. acnes* biofilm in degenerated disc tissue, as shown in Figures 1, 2 (Capoor et al., 2017), (iv) experimental data showing *P. acnes* induces disc degeneration in animal models (Dudli et al., 2016; Shan et al., 2017), and (v) the shared central role of IL-1β within acne vulgaris and DDD (Le Maitre et al., 2005; Wuertz and Haglund, 2013), we hypothesize, that a role for *P. acnes* in the etiology of DDD is biologically plausible, with IL-1β expression representing the driving mechanism behind its pathogenesis.

**Figure 3:**
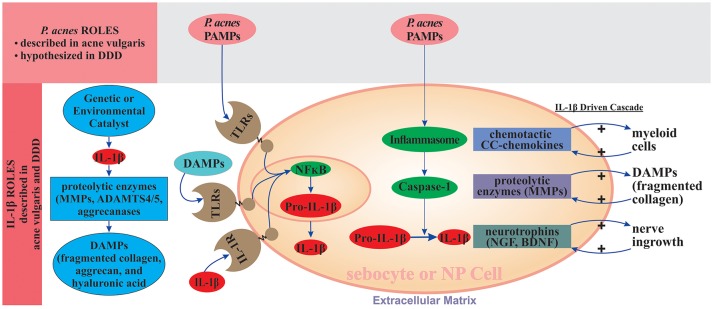
IL-1β driven degenerative state in acne vulgaris and degenerative disc disease. Following the initiating event, sebocytes and NP and AF cells promote production of IL-1β. IL-1β signaling facilitates extracellular matrix degradation through induction of proteolytic enzymes, including MMPs and ADAMTS4/5. *P. acnes* PAMPs and extracellular matrix degradation products DAMPs (e.g., fragmented collagen, aggrecan, and hyaluronic acid) bind to TLRs, which activate NF-κB, leading to the synthesis of pro-IL-1β; *P. acnes* PAMPs also activate inflammasomes (NLRP3) and caspase-1, which convert pro-IL-1β to IL-1β. IL-1β stimulates production of CC-chemokines that recruit myeloid cells and proteolytic matrix metalloproteases (MMPs) that degrade the ECM. Positive feedback loops arise when IL-1β binds to IL-1R and when extracellular matrix degradation products (DAMPs, e.g., fragmented collagen) activate TLRs. Both binding events stimulate IL-1β production. Inflammatory signaling is amplified since IL-1β is not only NF-κB target gene, but also its activator through binding to IL-R1. The nerve ingrowth into the degenerated disc, usually accompanied by presence of the annular fissures eventually herniation, is considered a main source of nociception and thus low back and/or radicular pain. IL-1β induces the expression of neurotrophins like NGF and BDNF supporting the nerve. ADAMTS-4/5, a disintegrin and metalloproteinase with thrombospondin motifs 4/5; AF, annulus fibrosus; BDNF, brain-derived neurotrophic factor; DAMPs, damage-associated molecular patterns; PAMPs, pathogen-associated molecular patterns; DDD, degenerative disc disease; ECM, extracellular matrix; IL-1R, interleukin-1 receptor; MMPs, matrix metalloproteinases; NF-κB, nuclear factor κB; NGF, nerve growth factor; NP, nucleus pulposus; TLRs, Toll-like receptors.

As shown in Figure 3, *P. acnes* can affect the initiation and/or progression of DDD by stimulating IL-1β synthesis through several potential routes: (i) *P. acnes* infection can promote IL-1β production by AF and NP cells through a direct interaction between *P. acnes*-specific PAMPs and TLR2 on the surfaces of NP and AF cells, which activates NF-κB signaling and leads to the production of pro-IL-1β; (ii) hyaluronidase released by *P. acnes* cleaves hyaluronic acid within the disc matrix to form short fragments with ability to activate NF-κB signaling leading to production of pro-IL-1β; (iii) *P. acnes*-specific PAMPs can also activate NLRP3-inflammasome and caspase-1, which can convert pro-IL-1β to mature IL-1β; (iv) the IL-1β produced through the action of *P. acnes* can amplify inflammatory signaling through interaction with IL-1R, which creates a positive feedback loop; and (v) the IL-1β produced can further unleash signaling pathways that lead to the production of MMPs and ADAMTS, which degrade the extracellular matrix, producing *P. acnes*-related DAMPs that can interact with AF, NP, and myeloid cells to further activate NF-κB signaling and produce a second positive feedback loop.

Since there are several independent routes by which *P. acnes* can promote the synthesis of IL-1β, and there is a positive feedback loop in the regulation of IL-1β levels, we propose that *P. acnes* is a strong contributor to IL-1β-based pathogenetic mechanisms that lead to DDD. We further hypothesize that if there are other etiological factors contributing to DDD through the promotion of IL-1β, *P. acnes* significantly amplifies the significance of this pre-existing IL-1β action and facilitates the pathogenesis in synergy with other etiological factors of DDD.

Furthermore, we propose that in some patients where no *P. acnes* is detected directly by culture- or non-culture methods within disc material, the central role of IL-1β may still be a “smoking gun” for an infection that may no longer be present but has created a state of chronic inflammation and bone destruction that still persists in some form.

Finally, complex inherited alterations in the inflammasome/ IL-1β system may fine-tune its functional capacity and cause an imbalance of pro- and anti-inflammatory responses that determine the individual risk, time of onset, and severity of *P. acnes*-associated DDD.

## Testing the hypothesis and implications for health care provision

Since there is strong experimental evidence of IL-1β involvement in the pathogenesis of DDD (Wuertz and Haglund, 2013; Risbud and Shapiro, 2014), our aim here is to propose experimental approaches to assess the ability and mechanism by which *P. acnes* promotes IL-1β synthesis and function, and not to prove its effects on DDD pathogenesis *per se*. NP and AF cells have been shown to express TLR2 (Le Maitre et al., 2005), and there is evidence of *P. acnes* interaction with TLR2 (Kistowska et al., 2014; Li et al., 2014). Therefore, the ability of *P. acnes* to induce IL-1β should be demonstrated by monitoring IL-1β levels in NP and AF cell cultures and *in vivo* models of DDD that have been infected with *P. acnes* strains isolated from degenerated human discs, and covering different phylogenetic groups that are known to vary in their inflammatory nature as well as other pathogenic characteristics (McDowell et al., 2013). Since *P. acnes* has been shown to form biofilm in DDD, its ability to promote IL-1β production should be compared with heavy biofilm formers and isolates that fail to actively form biofilms (Holmberg et al., 2009). Finally, clinical samples of degenerated disc tissue shown to be infected with *P. acnes* should be associated with IL-1β levels higher than those seen with non-infected degenerated discs.

The ability of *P. acnes* to activate TLR2 signaling in myeloid cells, especially macrophages, has been already demonstrated in acne vulgaris confirming another route of *P. acnes*-associated IL-1β production. Finally, short fragments of hyaluronic acid have been shown to interact with TLR2 present on NP cells (Quero et al., 2013), providing evidence for the *P. acnes*-associated IL-1β production, through hyaluronidase secretion. To provide a definitive test of the hypothesis that *P. acnes* is a strong contributor to IL-1β-based DDD pathogenesis, *in vitro* and *in vivo* experiments will be performed to quantitatively compare the ability of *P. acnes* to induce IL-1β with that of other known activators of IL-1β commonly used in DDD.

To identify the concrete mechanisms affecting the propensity of *P. acnes* to activate the various routes of IL-1β induction, the following experimental approaches could be used *in vitro* and in animal models: (i) a series of knock-in and knock-down experiments of the critically important genes linked to various mechanisms of IL-1β induction and functioning (e.g., TLR2, NLR3, MyD88 or caspase-1), (ii) experiments with a panel of inhibitors specifically affecting signaling pathways associated with the complex cascade of IL-1β processing and function, and finally (iii) experiments with models for which the signaling ligands are naturally or experimentally altered.

Evidence that *P. acnes* infection may incite a chronic inflammatory response that persists after the organism is no longer present could be investigated via serological studies and analysis of samples for bacterial products and signature metabolites that could still be perpetuating inflammation (Magnitsky et al., 2017), or via a series of model infections in which animals will be treated for *P. acnes* with antibiotics over a wide range of time-points post-infection and the grade of disc degeneration compared between the groups. A prior history of moderate-to-severe acne vulgaris may also be important in this context as has been shown in relation to the potential role of *P. acnes* in prostate cancer (Ugge et al., 2018).

Even if the hypothesis put forward here turns out to be incorrect or only partially correct, it will at least have served to stimulate more research on the potential role of *P. acnes* in DDD, which is vitally important because of the potential benefits for patients and wider society.

The main clinical presentation of DDD is chronic low back pain (CLBP). CLBP is one of the world's most debilitating conditions, presenting with substantial socio-economic and health-care consequences (Vos et al., 2012). Based on our theoretical concept, the causal therapy for patients with DDD associated with *P. acnes* infection is appropriate antibiotic treatment. However, even if *P. acnes* infection is successfully eradicated by antibiotic treatment, ongoing inflammatory processes with IL-1β playing the central role, along with other etiological factors, can cause treatment failure.

Since anti-IL-1β drugs have already been approved for the treatment of inflammatory and autoinflammatory diseases (Dinarello et al., 2012), therapeutic approaches targeting IL-1β in combination with appropriate antibiotic treatment, concomitantly or in sequence, should be considered as a novel treatment approach. Inhibition of IL-1β signaling can be via direct targeting of the molecule with biologics or via pro-drug inhibitors of the inflammasome that target caspase-1 or NLRP3 (Risbud and Shapiro, 2014). As an exemplar of this approach, treatment of patients with acne using the humanized anti-IL-1β antibody Gevokizumab demonstrated significant clinical response with reductions in inflammatory lesions (Fenini et al., 2017). While acknowledging the complicated nature of DDD, and the potential for different drivers of inflammation, there is already promising experimental evidence of the ability of IL-1β-targeted approaches to affect pathogenic mechanisms of the condition both *in vitro* and *in vivo*, promoting ECM repair and inhibiting disc degeneration (Gorth et al., 2012).

The prerequisite for successful CLBP treatment is an accurate diagnosis. From this perspective, diagnosis of *P. acnes* infection and direct evidence of IL-1β over-expression present a strategy to rationalize a more tailored treatment and management strategy for patients with DDD, which is essential in a growing era of precision/ stratified medicine.

## Author contributions

OS and MC conceptualized the hypothesis and drafted the initial manuscript. AM, HB, AR, SD-D, TF, and PL all contributed to writing and editing of the subsequent drafts and final version of the manuscript.

### Conflict of interest statement

MC, OS, and AR have stock ownership or options in DiscitisDx, Inc. MC and OS have filed several patent applications, which have been assigned to DiscitisDx, Inc. The remaining authors declare that the research was conducted in the absence of any commercial or financial relationships that could be construed as a potential conflict of interest.

## References

[B1] Agak G. W. Qin M. Nobe J. Kim M. H. Krutzik S. R. Tristan G. R.. (2014). *Propionibacterium acnes* induces an IL-17 response in acne vulgaris that is regulated by vitamin A and vitamin D. J. Invest. Dermatol. 134, 366–373. 10.1038/jid.2013.334 23924903 PMC4084940

[B2] Albert H. B. Sorensen J. S. Christensen B. S. Manniche C. (2013). Antibiotic treatment in patients with chronic low back pain and vertebral bone edema (Modic type 1 changes): a double-blind randomized clinical controlled trial of efficacy. Eur. Spine J. 22, 697–707. 10.1007/s00586-013-2675-y 23404353 PMC3631045

[B3] Birkenmaier C. (2013). Should we start treating chronic low back pain with antibiotics rather than with pain medications? Korean J. Pain 26, 327–335. 10.3344/kjp.2013.26.4.327 24155998 PMC3800704

[B4] Capoor M. N. Ruzicka F. Machackova T. Jancalek R. Smrcka M. Schmitz J. E.. (2016). Prevalence of *Propionibacterium acnes* in intervertebral discs of patients undergoing lumbar microdiscectomy: a prospective cross-sectional study. PLoS ONE 11:e0161676. 10.1371/journal.pone.0161676 27536784 PMC4990245

[B5] Capoor M. N. Ruzicka F. Schmitz J. E. James G. A. Machackova T. Jancalek R.. (2017). *Propionibacterium acnes* biofilm is present in intervertebral discs of patients undergoing microdiscectomy. PLoS ONE 12:e0174518. 10.1371/journal.pone.0174518 28369127 PMC5378350

[B6] Chen Z. H. Jin S. H. Wang M. Y. Jin X. L. Lv C. Deng Y. F.. (2015). Enhanced NLRP3, caspase-1, and IL- 1beta levels in degenerate human intervertebral disc and their association with the grades of disc degeneration. Anat. Rec. 298, 720–726. 10.1002/ar.23059 25284686

[B7] Coscia M. F. Denys G. A. Wack M. F. (2016). *Propionibacterium acnes*, coagulase-negative *Staphylococcus*, and the “Biofilm-like” intervertebral disc. Spine 41, 1860–1865. 10.1097/BRS.0000000000001909 27669046 PMC5158091

[B8] Denk F. Bennett D. L. McMahon S. B. (2017). Nerve growth factor and pain mechanisms. Annu. Rev. Neurosci. 40, 307–325. 10.1146/annurev-neuro-072116-031121 28441116

[B9] Dinarello C. A. Simon A. van der Meer J. W. (2012). Treating inflammation by blocking interleukin-1 in a broad spectrum of diseases. Nat. Rev. Drug Discov. 11, 633–652. 10.1038/nrd3800 22850787 PMC3644509

[B10] Dudli S. Liebenberg E. Magnitsky S. Miller S. Demir-Deviren S. Lotz J. C. (2016). *Propionibacterium acnes* infected intervertebral discs cause vertebral bone marrow lesions consistent with Modic changes. J. Orthop. Res. 34, 1447–1455. 10.1002/jor.23265 27101067

[B11] Fenini G. Contassot E. French L. E. (2017). Potential of IL-1, IL-18 and inflammasome inhibition for the treatment of inflammatory skin Diseases. Front. Pharmacol. 8:278. 10.3389/fphar.2017.00278 28588486 PMC5438978

[B12] Freemont A. J. Peacock T. E. Goupille P. Hoyland J. A. O'Brien J. Jayson M. I. (1997). Nerve ingrowth into diseased intervertebral disc in chronic back pain. Lancet 350, 178–181. 9250186 10.1016/s0140-6736(97)02135-1

[B13] Ganko R. Rao P. J. Phan K. Mobbs R. J. (2015). Can bacterial infection by low virulent organisms be a plausible cause for symptomatic disc degeneration? A systematic review. Spine 40, E587–E592. 10.1097/BRS.0000000000000832 25955094

[B14] Gorth D. J. Mauck R. L. Chiaro J. A. Mohanraj B. Hebela N. M. Dodge G. R.. (2012). IL-1ra delivered from poly(lactic-co-glycolic acid) microspheres attenuates IL-1beta-mediated degradation of nucleus pulposus *in vitro*. Arthritis Res. Ther. 14:R179. 10.1186/ar3932 22863285 PMC3580573

[B15] Gruber H. E. Hoelscher G. L. Bethea S. Hanley E. N. Jr. (2012). Interleukin 1-beta upregulates brain-derived neurotrophic factor, neurotrophin 3 and neuropilin 2 gene expression and NGF production in annulus cells. Biotech. Histochem. 87, 506–511. 10.3109/10520295.2012.703692 22853041

[B16] Hart L. G. Deyo R. A. Cherkin D. C. (1995). Physician office visits for low back pain. Frequency, clinical evaluation, and treatment patterns from a U.S. national survey. Spine 20, 11–19. 10.1097/00007632-199501000-00003 7709270

[B17] Holmberg A. Lood R. Mörgelin M. Söderquist B. Holst E. Collin M.. (2009). Biofilm formation by *Propionibacterium acnes* is a characteristic of invasive isolates. Clin. Microbiol. Infect. 15, 787–795. 10.1111/j.1469-0691.2009.02747.x 19392888

[B18] Jahns A. C. Lundskog B. Ganceviciene R. Palmer R. H. Golovleva I. Zouboulis C. C.. (2012). An increased incidence of *Propionibacterium acnes* biofilms in acne vulgaris: a case-control study. Br. J. Dermatol. 167, 50–58. 10.1111/j.1365-2133.2012.10897.x 22356121

[B19] Kistowska M. Gehrke S. Jankovic D. Kerl K. Fettelschoss A. Feldmeyer L.. (2014). IL-1beta drives inflammatory responses to propionibacterium acnes *in vitro* and *in vivo*. J. Invest. Dermatol. 134, 677–685. 10.1038/jid.2013.438 24157462

[B20] Lasigliè D. Traggiai E. Federici S. Alessio M. Buoncompagni A. Accogli A.. (2011). Role of IL-1 beta in the development of human T(H)17 cells: lesson from NLPR3 mutated patients. PLoS ONE 6:e20014. 10.1371/journal.pone.0020014 21637346 PMC3102666

[B21] Le Maitre C. L. Freemont A. J. Hoyland J. A. (2005). The role of interleukin-1 in the pathogenesis of human intervertebral disc degeneration. Arthritis Res. Ther. 7, R732–R745. 10.1186/ar1732 15987475 PMC1175026

[B22] Le Maitre C. L. Hoyland J. A. Freemont A. J. (2007). Interleukin-1 receptor antagonist delivered directly and by gene therapy inhibits matrix degradation in the intact degenerate human intervertebral disc: an *in situ* zymographic and gene therapy study. Arthritis Res. Ther. 9:R83. 10.1186/ar2282 17760968 PMC2206387

[B23] Li Z. J. Choi D. K. Sohn K. C. Seo M. S. Lee H. E. Lee Y.. (2014). *Propionibacterium acnes* activates the NLRP3 inflammasome in human sebocytes. J. Invest. Dermatol. 134, 2747–2756. 10.1038/jid.2014.221 24820890

[B24] Magnitsky S. Dudli S. Tang X. Kaur J. Diaz J. Miller S. (2017). Quantification of propionic acid in the bovine spinal disk after infection of the tissue with *P. Acnes* bacteria. Spine 43, E634–E638. 10.1097/BRS.0000000000002448 PMC5893447 29019804

[B25] McDowell A. Nagy I. Magyari M. Barnard E. Patrick S. (2013). The opportunistic pathogen *Propionibacterium acnes*: insights into typing, human disease, clonal diversification and CAMP factor evolution. PLoS ONE 8:e70897. 10.1371/journal.pone.0070897 24058439 PMC3772855

[B26] Moradi Tuchayi S. Makrantonaki E. Ganceviciene R. Dessinioti C. Feldman S. R. Zouboulis C. C. (2015). Acne vulgaris. Nat. Rev. Dis. Primers 1:15029. 10.1038/nrdp.2015.29 27189872

[B27] Nazipi S. Stødkilde-Jørgensen K. Scavenius C. Bruggemann H. (2017). The Skin bacterium *Propionibacterium acnes* employs two variants of hyaluronate lyase with distinct properties. Microorganisms 5:57. 10.3390/microorganisms5030057 28895889 PMC5620648

[B28] Quero L. Klawitter M. Schmaus A. Rothley M. Sleeman J. Tiaden A. N.. (2013). Hyaluronic acid fragments enhance the inflammatory and catabolic response in human intervertebral disc cells through modulation of toll-like receptor 2 signalling pathways. Arthritis Res. Ther. 15:R94. 10.1186/ar4274 23968377 PMC3978638

[B29] Risbud M. V. Shapiro I. M. (2014). Role of cytokines in intervertebral disc degeneration: pain and disc content. Nat. Rev. Rheumatol. 10, 44–56. 10.1038/nrrheum.2013.160 24166242 PMC4151534

[B30] Sanford J. A. Zhang L. J. Williams M. R. Gangoiti J. A. Huang C. M. Gallo R. L. (2016). Inhibition of HDAC8 and HDAC9 by microbial short-chain fatty acids breaks immune tolerance of the epidermis to TLR ligands. Sci. Immunol. 1:eaah4609. 10.1126/sciimmunol.aah4609 28783689

[B31] Sardana K. Verma G. (2017). *Propionibacterium acnes* and the Th1/Th17 axis, implications in acne pathogenesis and treatment. Indian J. Dermatol. 62, 392–394. 10.4103/ijd.IJD_483_16 28794550 PMC5527720

[B32] Shaheen B. Gonzalez M. (2011). A microbial aetiology of acne: what is the evidence? Br. J. Dermatol. 165, 474–485. 10.1111/j.1365-2133.2011.10375.x 21495996

[B33] Shan Z. Zhang X. Li S. Yu T. Liu J. Zhao F. (2017). *Propionibacterium acnes* incubation in the discs can result in time-dependent modic changes: a long-term rabbit model. Spine 42, 1595–1603. 10.1097/BRS.0000000000002192 28399545

[B34] Stirling A. Worthington T. Rafiq M. Lambert P. A. Elliott T. S. (2001). Association between sciatica and *Propionibacterium acnes*. Lancet 357, 2024–2025. 10.1016/S0140-6736(00)05109-6 11438138

[B35] Ugge H. Udumyan R. Carlsson J. Andrén O. Montgomery S. Davidsson S.. (2018). Acne in late adolescence and risk of prostate cancer. Int. J. Cancer 142, 1580–1585. 10.1002/ijc.31192 29205339 PMC5838533

[B36] Unna P. G. (1895). The histological pathology of diseases of the skin. Br. J. Dermatol. 7, 83–85. 10.1111/j.1365-2133.1895.tb16839.x

[B37] Urquhart D. M. Zheng Y. Cheng A. C. Rosenfeld J. V. Chan P. Liew S.. (2015). Could low grade bacterial infection contribute to low back pain? A systematic review. BMC Med. 13:13. 10.1186/s12916-015-0267-x 25609421 PMC4320560

[B38] Vos T. Flaxman A. D. Naghavi M. Lozano R. Michaud C. Ezzati M.. (2012). Years lived with disability (YLDs) for 1160 sequelae of 289 diseases and injuries 1990-2010: a systematic analysis for the Global Burden of Disease Study 2010. Lancet 380, 2163–2196. 10.1016/S0140-6736(12)61729-2 23245607 PMC6350784

[B39] Wang J. Tian Y. Phillips K. L. Chiverton N. Haddock G. Bunning R. A.. (2013). Tumor necrosis factor alpha- and interleukin-1beta-dependent induction of CCL3 expression by nucleus pulposus cells promotes macrophage migration through CCR1. Arthritis Rheum. 65, 832–842. 10.1002/art.37819 23233369 PMC3582738

[B40] Wuertz K. Haglund L. (2013). Inflammatory mediators in intervertebral disk degeneration and discogenic pain. Global Spine J. 3, 175–184. 10.1055/s-0033-1347299 24436868 PMC3854585

[B41] Yang W. Yu X. H. Wang C. He W. S. Zhang S. J. Yan Y. G.. (2015). Interleukin-1beta in intervertebral disk degeneration. Clin. Chim. Acta 450, 262–272. 10.1016/j.cca.2015.08.029 26341894

